# Exosomal circ-BRWD1 contributes to osteoarthritis development through the modulation of miR-1277/TRAF6 axis

**DOI:** 10.1186/s13075-021-02541-8

**Published:** 2021-06-03

**Authors:** Zhenye Guo, Huan Wang, Feng Zhao, Min Liu, Feida Wang, Mingming Kang, Weifu He, Zhi Lv

**Affiliations:** 1 Department of Orthopaedics, Second Hospital of Shanxi Medical University, No.2 Hospital, Shanxi Medical University, 382 Wuyi Road, Xinghualing District, Taiyuan City, Shanxi Province China; 2 Department of Orthopaedics, Shanxi Medical University, West Hospital, Second Hospital, Shanxi Medical University, 379 Yingzhi Street, Wanbai District, Taiyuan City, Shanxi Province China

**Keywords:** OA, Exosome, Circ-BRWD1, MiR-1277, TRAF6

## Abstract

**Background:**

Circular RNAs (circRNAs) can act as vital players in osteoarthritis (OA). However, the roles of circRNAs in OA remain obscure. Herein, we explored the roles of exosomal circRNA bromodomain and WD repeat domain containing 1(circ-BRWD1) in OA pathology.

**Methods:**

*In vitro* model of OA was constructed by treating CHON-001 cells with interleukin-1β (IL-1β). Quantitative real-time polymerase chain reaction (qRT-PCR) assay was used for circ-BRWD1, BRWD, miR-1277, and TNF receptor-associated factor 6 (TRAF6) levels. RNase R assay was conducted for the feature of circ-BRWD1. Transmission electron microscopy (TEM) was employed to analyze the morphology of exosomes. Western blot assay was performed for protein levels. Cell Counting Kit-8 (CCK-8) assay, flow cytometry analysis, and 5-Ethynyl-2′-deoxyuridine (EDU) assay were adopted for cell viability, apoptosis, and proliferation, respectively. Enzyme-linked immunosorbent assay (ELISA) was carried out for the concentrations of interleukin-6 (IL-6) and interleukin-8 (IL-8). Dual-luciferase reporter and RNA immunoprecipitation (RIP) assays were used to analyze the interaction between miR-1277 and circ-BRWD1 or TRAF6.

**Results:**

Circ-BRWD1 was increased in OA cartilage tissues, IL-1β-treated CHON-001 cells, and the exosomes derived from IL-1β-treated CHON-001 cells. Exosome treatment elevated circ-BRWD1 level, while exosome blocker reduced circ-BRWD1 level in IL-1β-treated CHON-001 cells. Silencing of circ-BRWD1 promoted cell viability and proliferation and repressed apoptosis, inflammation, and extracellular matrix (ECM) degradation in IL-1β-stimulated CHON-001 cells. For mechanism analysis, circ-BRWD1 could serve as the sponge for miR-1277 to positively regulate TRAF6 expression. Moreover, miR-1277 inhibition ameliorated the effects of circ-BRWD1 knockdown on IL-1β-mediated CHON-001 cell damage. Additionally, miR-1277 overexpression relieved IL-1β-induced CHON-001 cell injury, while TRAF6 elevation restored the impact.

**Conclusion:**

Exosomal circ-BRWD1 promoted IL-1β-induced CHON-001 cell progression by regulating miR-1277/TRAF6 axis.

## Highlights

1. Circ-BRWD1 is upregulated in osteoarthritis patients and IL-1β-induced chondrocyte cell line.
2. Exosomal circ-BRWD1 is upregulated in IL-1β-induced chondrocyte cell line.
3. Circ-BRWD1 knockdown promotes IL-1β-mediated chondrocyte cell viability and inhibits apoptosis, inflammation, and ECM degradation.
4. Circ-BRWD1 knockdown ameliorates IL-1β-mediated chondrocyte injury by regulating miR-1277/TRAF6 axis.

## Introduction

Osteoarthritis (OA) is a slow-developing degenerative joint disease with a high incidence, which is related to age, obesity, tension, strain, trauma, joint deformities, and other factors [1, 2]. OA is characterized by structural change of subchondral bone, inflammation of synovitis, and destruction of cartilage matrix [3, 4]. At present, methods such as anti-inflammatory analgesics and artificial joint replacement surgery are mainly used to reduce OA patient’s joint pain and control its progress; however, the side effects and high costs hinder their wide application [5, 6]. Thus, identifying novel treatment strategies for OA is very necessary.

Circular RNAs (circRNAs) are a sort of non-coding RNAs (ncRNAs) with closed-loop structures, which can regulate gene expression by competitive targeting microRNAs (miRNAs) [7, 8]. Emerging evidence has reported that circRNAs function as critical regulators in multiple human diseases, including OA. For example, circ_00050105 facilitated ECM degradation and inflammatory response in interleukin-1β (IL-1β)-treated chondrocytes by sponging miR-26a [9]. Circ_0045714 accelerated chondrocyte proliferation and ECM synthesis and restrained apoptosis by modulating miR-193b and IGF1R [10]. As for circ-BRWD1 (circ_0116061), the heatmap showed it was upregulated in OA cartilage tissues compared to normal cartilage tissues [11]. However, whether the abnormal expression of circ-BRWD1 (circ_0116061) plays a role in OA is still undefined. Exosomes are tiny particles with a diameter of 30–150 nm which can be released by multiple cell types [12]. It has been documented that circRNAs are abundant in exosomes and can be transferred into other cells to regulate biological functions [13]. In this study, the functions of exosome-mediated circ-BRWD1 in OA were investigated.

As a class of small ncRNAs, miRNAs participate in regulating multiple biological processes via recognization of the 3′ untranslated region (3′UTR) of target mRNAs [14]. In OA, miR-34a overexpression facilitated the apoptosis and suppressed the proliferation of chondrocytes in the pathophysiological process of OA via targeting SIRT1/p53 signaling pathway [15]. MiR-101 inhibition reversed IL-1β-activation ECM degradation in chondrocytes through targeting Sox9 [16]. Moreover, Wang et al. disclosed that miR-1277 alleviated ECM degradation in IL-1β-treated articular chondrocytes via interacting with MMP13 and ADAMTS5 [17]. These findings indicated the vital role of miR-1277 in OA. TNF receptor-associated factor 6 (TRAF6) has been demonstrated to be targeted by miR-146a to regulate OA chondrocyte proliferation and apoptosis [18]. By analyzing bioinformatics software circinteractome and Targetscan, miR-1277 was found to contain the binding sites of circ-BRWD1 and TRAF6, but the relationships among circ-BRWD1, miR-1277, and TRAF6 in OA development are still unclear.

The present study aimed to determine the expression profiles of exosomal circ-BRWD1, miR-1277, and TRAF6 in IL-1β-activated chondrocytes and further explore their functional roles in OA development.

## Materials and methods

### Tissues acquisition

The OA cartilage tissue specimens were harvested from 32 OA patients undergoing total knee arthroplasty and the normal cartilage tissue specimens were harvested from 32 traumatic amputees at the Second Hospital of Shanxi Medical University. The specimens were preserved at − 80 °C until further experiments. The work was approved by the Ethics Committee of Second Hospital of Shanxi Medical University. Written informed consents were provided by all patients.

### Cell culture and IL-1β treatment

The chondrocyte cell line CHON-001 was acquired from the American Type Culture Collection (ATCC, Manassas, VA, USA) and maintained in Dulbecco’s modified Eagle’s medium (DMEM; Sigma-Aldrich, St. Louis, MO, USA) added with 10% fetal bovine serum (FBS; Sigma-Aldrich) and 1% penicillin-streptomycin (Sigma-Aldrich) in a humid incubator with 5% CO_2_ at 37 °C.

To stimulate OA chondrocyte model, CHON-001 cells were exposed to IL-1β (5 ng/mL, 10 ng/mL, and 20 ng/mL; Sigma-Aldrich) at 37 °C for 24 h. The untreated cells (0 ng/mL) were used as controls. 10 ng/mL IL-1β-treated cells were chosen for further experiments.

### Quantitative real-time polymerase chain reaction (qRT-PCR)

The RNA extraction was conducted through the usage of TRIzol reagent (Beyotime, Shanghai, China). RNA treatment was performed on total RNA with 3 U/μg RNase R (Epicenter Biotechnologies, Madison, WI, USA) for 15 min at 37 °C. Then the M-MLV Reverse Transcriptase Kit (Promega, Madison, WI, USA) or TaqMan MicroRNA Reverse Transcription Kit (Applied Biosystems, Foster City, CA, USA) was adopted for the reverse transcription of RNA samples. QRT-PCR analysis was manipulated with BeyoFast™ SYBR Green qPCR Mix (Beyotime) and indicated primers (GeneCopoeia, Guangzhou, China). The primers were circ-BRWD1: (F: 5′-AGCACGGATTTGGAGATTTG-3′ and R: 5′-CGTAGCAAAGACTGCCTTCC-3′); BRWD1: (F: 5′-CCAGCGCATCGGTCCTATG-3′ and R: 5′-CTTCCTGCACCAAGTAAAGAAGT-3′); miR-1277: (F: 5′-ACACTCCAGCTGGGAAATATATATATATATGT-3′ and R: 5′-TGGTGTCGTGGAGTCG-3′); TRAF6: (F: 5′-TGTTGCAGCAGCTATTTTGC-3′ and R: 5′-CTTCTCGAGGGCACTAGCAC-3′); GAPDH: (F: 5′-GGAAGGTGAAGGTCGGAGTC-3′ and R: 5′-CGTTCTCAGCCTTGACGGT-3′); U6: (F: 5′-GCTCGCTTCGGCAGCACATA-3′ and R: 5′-ACGCTTCACGAATTTGCGT-3′). The expression was estimated via the 2^-ΔΔCt^ strategy with GAPDH or U6 as a negative control.

### Subcellular fraction assay

The isolation of nuclear and cytoplasmic fractions was conducted with the PARIS Kit (Life Technologies, Grand Island, NY, USA) in line with the protocols of the manufacturers. The RNAs isolated from the fractions of CHON-001 cells were subjected to the aforementioned qRT-PCR analysis for circ-BRWD1, U6 (a control for nuclear fraction) and GAPDH (a control for cytoplasmic fraction) levels.

### Isolation of exosomes

The ExoQuick precipitation kit (System Biosciences, Mountain View, CA, USA) was used to isolate exosomes from the culture media of CHON-001 cells. Briefly, the media were collected and centrifuged at 1000×*g* for 10 min to sediment the cells. Then, the media were centrifuged at 10,000×*g* for 30 min to remove the dead cells and cellular debris. After that, the ExoQuick solution was supplemented into the supernatant for 30 min at 4 °C and then centrifuged for 40 min at 2000×*g*. Subsequently, the supernatant was removed and the exosome-containing pellet was washed with PBS (Beyotime) and then resuspended in PBS (Beyotime).

### Transmission electron microscopy (TEM)

The exosomal morphology was analyzed by TEM (JEOL Ltd., Tokyo, Japan) using negative staining according to the previous report [19]. The images were observed using the FEI TecnaiG2 spirit transmission electron microscope (Thermo-Fischer, Waltham, MA, USA) operated at 80 kV.

### Western blot assay

The extraction of total protein was done utilizing RIPA buffer (Beyotime) and quantified utilizing the BCA protein assay kit (Tiangen, Beijing, China). An equal amount of protein was resolved with sodium dodecyl sulfonate-polyacrylamide gel (Solarbio, Beijing, China) and then transferred onto polyvinylidene difluoride membranes (Sigma-Aldrich). After blocking in 5% defatted milk for 1 h at indoor temperature, the membranes were incubated with primary antibodies against CD9 (ab223052; Abcam, Cambridge, MA, USA), CD63 (ab68418; Abcam), CyclinD1 (ab226977; Abcam), Bax (ab104156; Abcam), matrix metalloprotein 13 (MMP13; ab39012; Abcam), aggrecan (ab36861; Abcam), TRAF6 (ab137452; Abcam) or GAPDH (ab37168; Abcam) overnight at 4 °C and indicated secondary antibody (ab6789; Abcam) for 1.5 h at indoor temperature. The ECL kit (Beyotime) was employed for chemiluminescence imaging.

### Exosomes or GW4869 treatment

CHON-001 cells were plated into 6-well plates (1 × 10^5^ cells/well) and then the exosomes (20 μg) derived from 10 ng/mL IL-1β-triggered CHON-001 cells were added into the culture media for 48 h. The control media were treated with PBS (Beyotime).

To block exosome secretion, IL-1β-treated CHON-001 cells were treated with 20 μM GW4869 (Sigma-Aldrich) for 2 h prior to IL-1β (Sigma-Aldrich) exposure. After 48 h, the supernatants were harvested for circ-BRWD1 expression via qRT-PCR analysis.

### Cell transfection

Circ-BRWD1 short interfering RNA (si-circ-BRWD1) and scramble control (si-NC), the overexpression vector of circ-BRWD1 (circ-BRWD1) and its control (pCD5-ciR), miR-1277 mimics (miR-1277) and control mimics (miR-NC), miR-1277 inhibitors (anti-miR-1277) and anti-miR-NC, the overexpression vector of TRAF6 (TRAF6) and pcDNA were purchased from GeneCopoeia. CHON-001 cells (1 × 10^4^ cells/well) were seeded into 24-well plates and the oligonucleotides (50 nM) or vectors (2 μg) were transfected into CHON-001 cells utilizing Lipofectamine 2000 (Invitrogen, Carlsbad, CA, USA) according to the manufacturers’ instructions.

### Cell Counting Kit-8 (CCK-8) assay

Following relevant treatment, CHON-001 cell viability was tested by CCK-8 assay. In brief, CHON-001 cells were plated into 96-well plates (1 × 10^4^ cells/well) and then cultivated for 72 h. 20 μL CCK-8 (Sigma-Aldrich) was treated into each well for further 3 h. After that, the OD value was measured at 450 nm with a microplate reader (BioTek, Winooski, VT, USA).

### Flow cytometry analysis

CHON-001 cell apoptosis was assessed with Annexin V-fluorescein isothiocyanate (FITC)/propidium iodide (PI) Apoptosis Detection Kit (Beyotime) according to the manufacturers’ guidelines. Briefly, CHON-001 cells were collected and rinsed with PBS (Beyotime) after relevant treatment and then resuspended in binding buffer. After that, 5 μL AnnexinV-FITC and 5 μL PI were added and maintained for 15 min in the dark. At last, cell apoptosis was examined with a FACScan® flow cytometry (BD Biosciences, San Jose, CA, USA).

### 5-Ethynyl-2′-deoxyuridine (EDU) assay

EDU assay was conducted to evaluate cell viability through the usage of EDU assay kit (Solarbio, Beijing, China). In short, the treated CHON-001 cells were seeded into 12-well plates and incubated with EDU buffer for 2 h at 37 °C. Then the cells were fixed with 4% formaldehyde for 30 min, permeabilizated for 20 min using 0.1% Triton X-100. Thereafter, the cells were exposed to EDU solution for 30 min and then stained cell nuclei using 5 μg/mL Hoechst 33342 for 20 min. The images were captured with a fluorescence microscope (Olympus, Tokyo, Japan) and the EDU-positive cells were counted.

### Enzyme-linked immunosorbent assay (ELISA)

The concentrations of interleukin-6 (IL-6) and interleukin-8 (IL-8) were conducted utilizing Human IL-6 ELISA Kit (ab178013; Abcam) and Human IL-8 ELISA Kit (ab214030; Abcam) according to the manufacturers’ instructions.

### Dual-luciferase reporter assay

The regions of circ-BRWD1 (or TRAF6 3′UTR) including or lacking the predicted miR-1277 binding sequences were amplified and inserted into pmirGLO vector (Promega), generating WT-circ-BRWD1, MUT-circ-BRWD1, TRAF6 3′UTR-WT, and TRAF6 3′UTR-MUT. The generation of WT-circ-BRWD1, MUT-circ-BRWD1, TRAF6 3′UTR-WT, and TRAF6 3′UTR-MUT was accomplished by GeneCopoeia. Then the generated vectors were transfected into CHON-001 cells in combination with miR-1277 or miR-NC. After the post-transfection for 48 h, dual-luciferase assay kit (Promega) was adopted for renilla and firefly luciferase activities.

### RNA immunoprecipitation (RIP) assay

The Magna RIP^TM^ RNA Binding Protein Immunoprecipitation Kit (Millipore, Bedford, MA, USA) was adopted for RIP assay. CHON-001 cells were lysed in RIP buffer and then incubated overnight with beads coated with antibody immunoglobulin G (IgG) or argonaute-2 (anti-Ago2) at 4 °C. Then the RNAs on the beads were retrieved for circ-BRWD1, miR-1277, and TRAF6 levels via qRT-PCR assay.

### Statistical analysis

The experiments were carried out in triple times. The collected data were analyzed by GraphPad Prism 7 software and presented as mean ± standard deviation. The linear correlation between the levels of miR-1277 and circ-BRWD1 or TRAF6 was estimated by Pearson’s correlation coefficient analysis. The differences between two groups were estimated by Student’s *t* test, while those among three groups were estimated by one-way analysis of variance (ANOVA). The differences were defined as significant when *P* < 0.05.

## Results

### Circ-BRWD1 was upregulated in OA cartilage tissues and IL-1β-induced CHON-001 cells

**Fig. 1:**
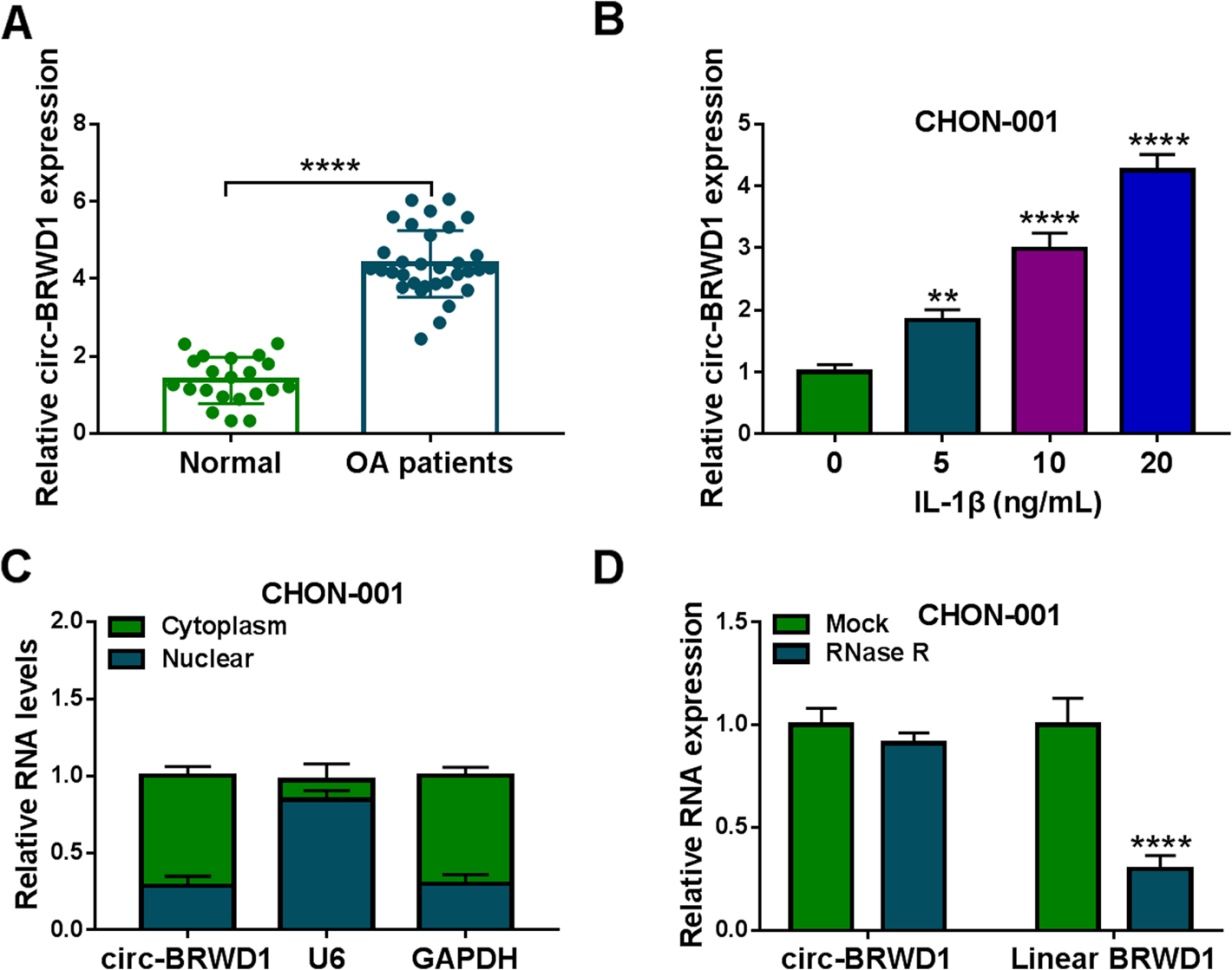
High level of circ-BRWD1 in OA cartilage tissues and IL-1β-induced CHON-001 cells. **A** QRT-PCR assay was conducted for circ-BRWD1 level in OA and non-OA cartilage tissues. **B** QRT-PCR assay was employed to determine circ-BRWD1 level in different doses of IL-1β-treated CHON-001 cells. **C** QRT-PCR assay was used for the expression of circ-BRWD1 in the nuclear and cytosolic fractions of CHON-001 cells. **D** QRT-PCR assay was used to determine the expression levels of circ-BRWD1 and linear BRWD1 in CHON-001 cells treated with or without RNase R. The expression of circ-BRWD1 and BRWD1 was examined by 2^-ΔΔCt^ method with normalization to GAPDH. **P < 0.001, ****P < 0.0001

At the beginning, qRT-PCR assay was conducted to detect the expression level of circ-BRWD1 in OA cartilage specimens and normal cartilage specimens. The results showed that circ-BRWD1 level was markedly increased in OA cartilage specimens compared to normal controls (Fig. 1A). Then we examined the level of circ-BRWD1 in IL-1β-stimulated CHON-001 cells and found that circ-BRWD1 level was enhanced in IL-1β-induced CHON-001 cells in a dose-dependent way (Fig. 1B). As demonstrated by subcellular fraction assay, circ-BRWD1 was mainly enriched in the cytoplasm of CHON-001 cells (Fig. 1C). Moreover, our results exhibited that circ-BRWD1 was resistant to RNase R treatment, while linear BRWD1 was digested by RNase R (Fig. 1D). These findings suggested that circ-BRWD1 might play a role in OA progression.

### Exosomal circ-BRWD1 level was elevated in IL-1β-induced CHON-001 cells

**Fig. 2:**
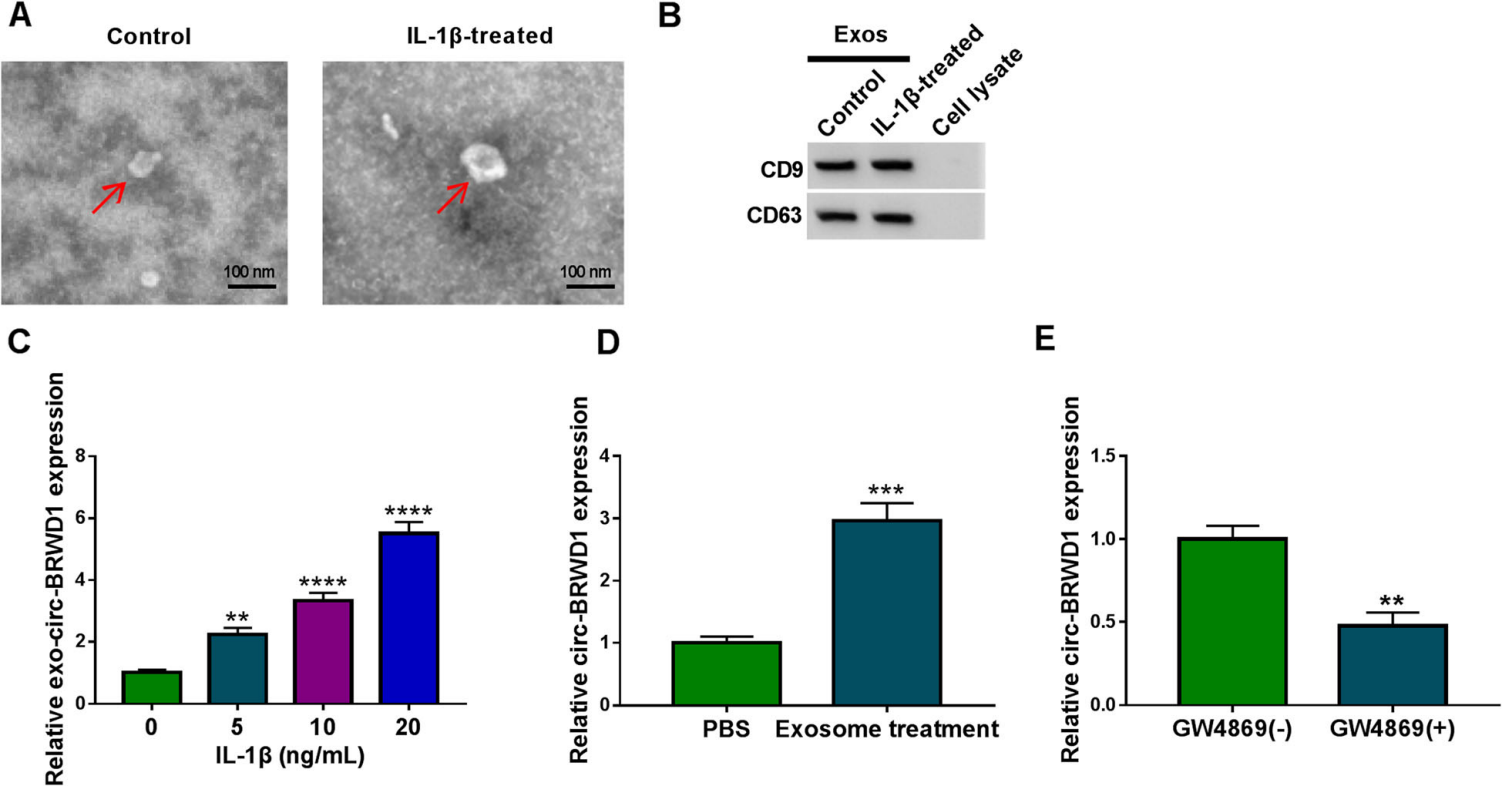
Circ-BRWD1 level was increased in IL-1β-treated CHON-001 cells-derived exosomes. **A** The morphology of isolated particles was analyzed using TEM. **B** The protein levels of CD9 and CD63 were measured by western blot assay. **C** The level of circ-BRWD1 in the exosomes derived from IL-1β-treated CHON-001 cells was examined by qRT-PCR assay. **D** Exosomes derived from IL-1β-treated CHON-001 cells were added into CHON-001 cells and then circ-BRWD1 level in CHON-001 cells was determined by qRT-PCR assay. **E** After IL-1β-treated CHON-001 cells were treated with or without GW4869 for 2 h and then exposed to IL-1β, the expression level of circ-BRWD1 was determined by qRT-PCR assay. The expression of circ-BRWD1 was examined by 2^-ΔΔCt^ method with normalization to GAPDH. **P < 0.01, ***P < 0.001, ****P < 0.0001

Subsequently, the exosomes were isolated from CHON-001 cells and IL-1β-treated CHON-001 cells. The morphology of the isolated exosomes was observed by TEM. The results indicated that the particles showed a round or oval membrane (Fig. 2A). Furthermore, we found that exosomal markers (CD9 and CD63) were highly expressed in the particles derived from CHON-001 cells and IL-1β-treated CHON-001 cells, as measured by western blot assay (Fig. 2B). These findings indicated that the isolated particles were exosomes. Next, the level of circ-BRWD1 in the exosomes derived from IL-1β-treated CHON-001 cells was examined by qRT-PCR assay, exhibiting that exosomal circ-BRWD1 level was elevated in IL-1β-treated CHON-001 cells dose-dependently (Fig. 2C). We then added the exosomes derived from IL-1β-treated CHON-001 cells into CHON-001 cells for 48 h and found the expression level of circ-BRWD1 released from exosomes was increased in CHON-001 cells compared to control groups (Fig. 2D). In addition, GW4869 was used to inhibit the exosome release by IL-1β-treated CHON-001 cells. The results showed that GW4869 treatment decreased the level of circ-BRWD1 in IL-1β-treated CHON-001 cells (Fig. 2E). All these results indicated that exosome mediated the transmission of circ-BRWD1 in IL-1β-treated CHON-001 cells.

### Circ-BRWD1 silencing reversed the effects on cell viability, apoptosis, inflammation, and ECM degradation in CHON-001 cells mediated by IL-1β

**Fig. 3:**
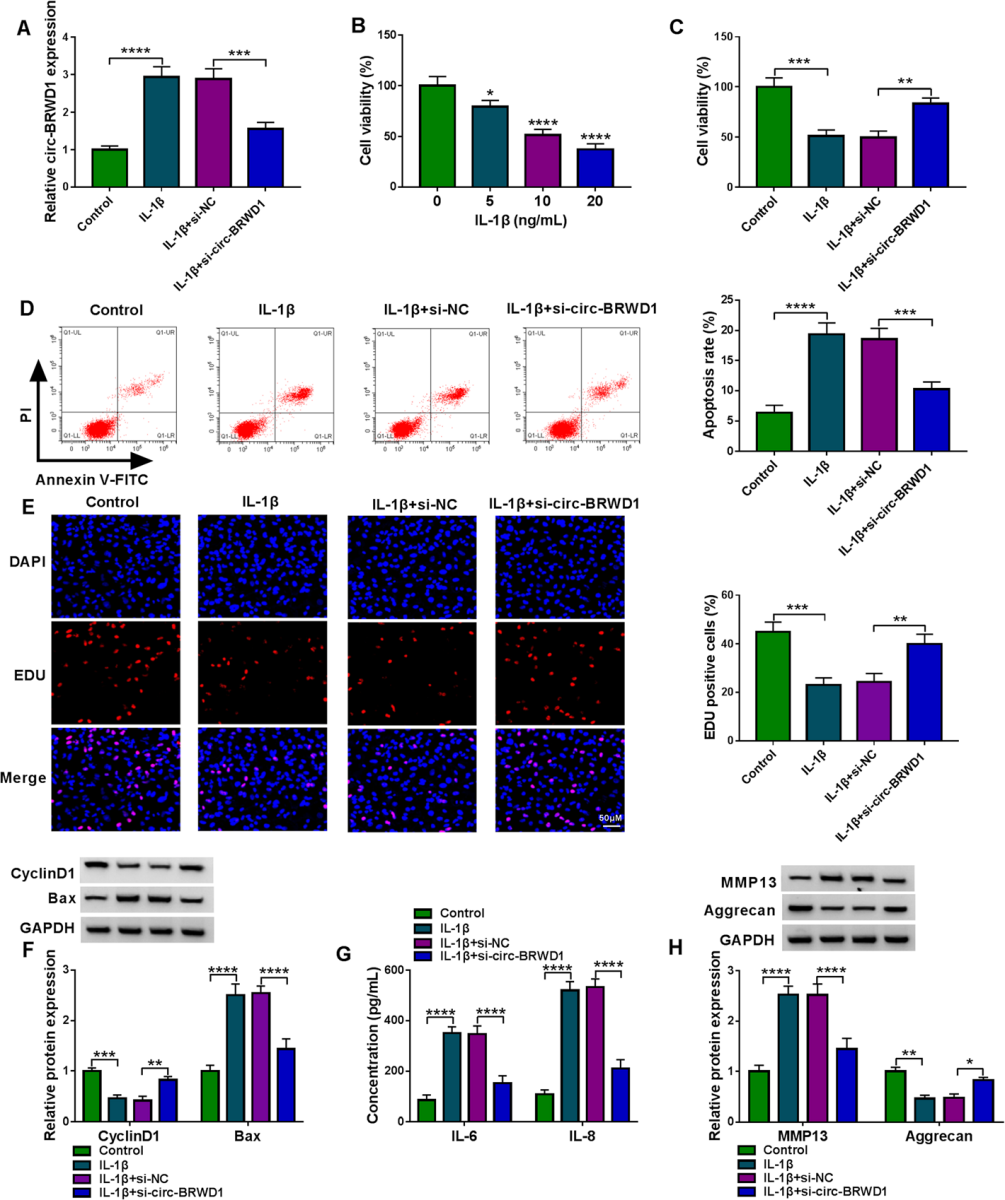
Knockdown of circ-BRWD1 reversed IL-1β-mediated cell viability, apoptosis, inflammation and ECM degradation in CHON-001 cells. **A** The expression level of circ-BRWD1 in IL-1β, IL-1β+si-NC, or IL-1β+si-circ-BRWD1 treated CHON-001 cells or untreated cells (control) was determined by qRT-PCR assay. **B** The viability of CHON-001 cells in different doses of IL-1β-treated CHON-001 cells was evaluated by CCK-8 assay. **C–F** CHON-001 cells were assigned to 4 groups: control, IL-1β, IL-1β+si-NC, and IL-1β+si-circ-BRWD1. **C**, **D** The viability and apoptosis of CHON-001 cells were analyzed by CCK-8 assay and flow cytometry analysis, respectively. **E** The proliferation of CHON-001 cells was assessed by western blot assay. **F** The protein levels of CyclinD1 and Bax in CHON-001 cells were measured by western blot assay. **G** The concentrations of IL-6 and IL-8 in CHON-001 cells were examined by ELISA. **H** The protein levels of MMP13 and aggrecan in CHON-001 cells were measured by western blot assay. The expression of circ-BRWD1 was examined by 2^-ΔΔCt^ method with normalization to GAPDH. *P < 0.05, **P < 0.01, ***P < 0.001, ****P < 0.0001

In order to investigate the functional roles of circ-BRWD1 in OA development, CHON-001 cells were transfected with si-circ-BRWD1 or si-NC and then treated with IL-1β for 24 h. As exhibited in Fig. 3A, the upregulation of circ-BRWD1 in CHON-001 cells caused by IL-1β treatment was effectively overturned by the transfection of si-circ-BRWD1, indicating the successful transfection of si-circ-BRWD1. CCK-8 assay showed that IL-1β treatment led to an obvious inhibition in the viability of CHON-001 cells in a concentration-dependent manner (Fig. 3B). CCK-8 assay also exhibited that circ-BRWD1 silencing reversed the inhibitory effect of IL-1β on cell viability in CHON-001 cells (Fig. 3C). Flow cytometry analysis showed that IL-1β treatment apparently facilitated the apoptosis of CHON-001 cells, whereas circ-BRWD1 deficiency abrogated the impact (Fig. 3D). EDU assay indicated that IL-1β inhibited CHON-001 cell proliferation, while circ-BRWD1 silencing reversed the effect (Fig. 3E). IL-1β treatment reduced CyclinD1 level and increased Bax level in CHON-001 cells, while circ-BRWD1 deficiency reversed the effects (Fig. 3F). The results of ELISA showed that IL-1β exposure markedly increased the levels of IL-6 and IL-8 in CHON-001 cells, while the effects were ameliorated by circ-BRWD1 inhibition (Fig. 3G). In addition, western blot assay showed that IL-1β treatment increased MMP13 level and decreased aggrecan level in CHON-001 cells, with circ-BRWD1 knockdown reversing the impacts (Fig. 3H). All these data suggested that circ-BRWD1 knockdown promoted IL-1β-mediated cell viability and suppressed IL-1β-mediated apoptosis, inflammation and ECM degradation in CHON-001 cells.

### MiR-1277 functioned as the sponge for circ-BRWD1

**Fig. 4:**
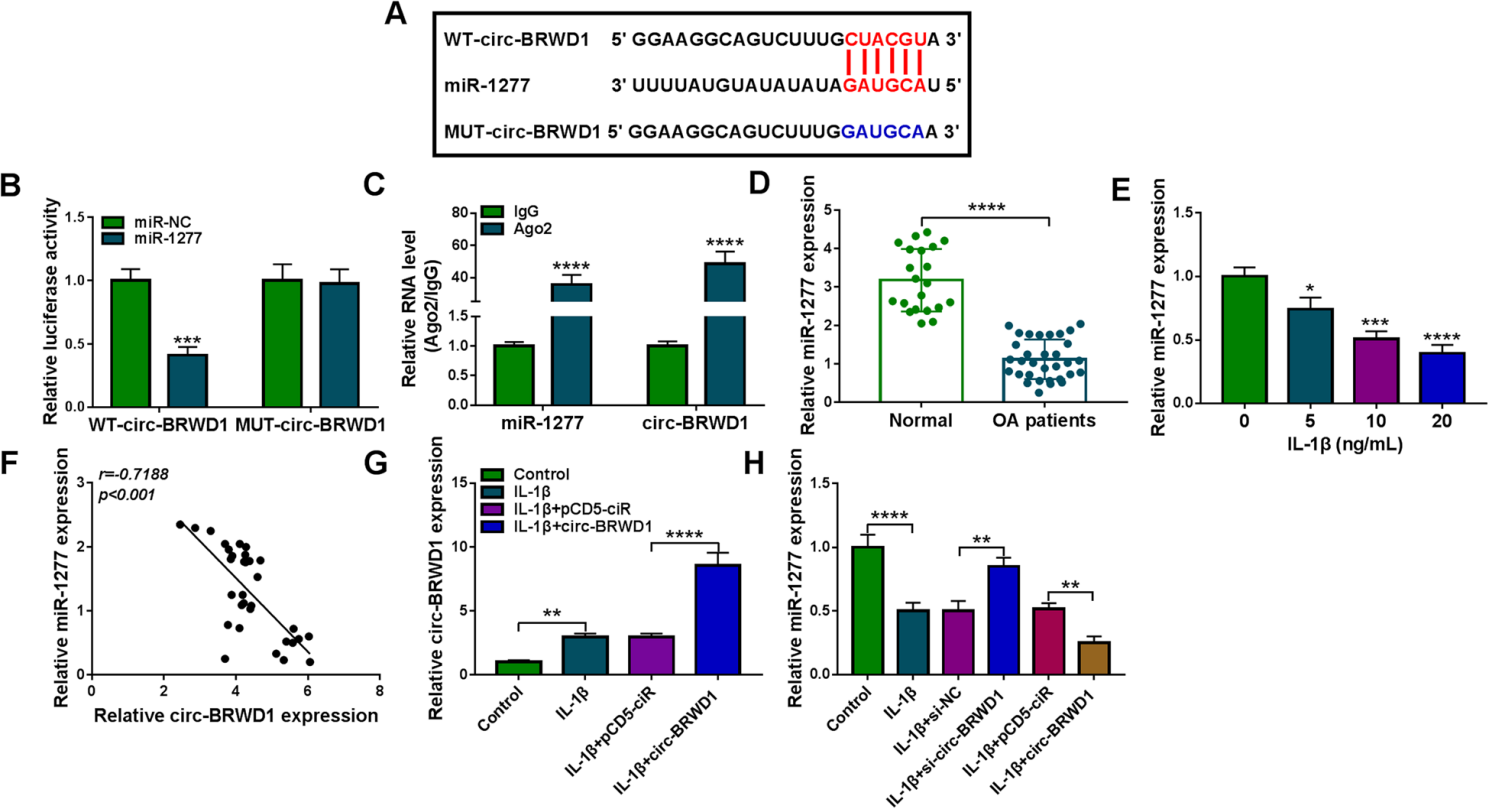
Circ-BRWD1 directly targeted miR-1277 and negatively regulated miR-1277 expression in IL-1β-stimulated CHON-001 cells. **A** The predicted binding sites between circ-BRWD1 and miR-1277. **B** The luciferase activity in CHON-001 cells co-transfected with miR-NC/miR-1277 and WT-circ-BRWD1/MUT-circ-BRWD1 was measured by dual-luciferase reporter assay. **C** The enrichment of miR-1277 and circ-BRWD1 in CHON-001 cells was determined by qRT-PCR assay following RIP assay. **D**, **E** The expression level of miR-1277 in OA cartilage tissues and IL-1β-stimulated CHON-001 cells was examined by qRT-PCR assay. **F** The linear correlation between circ-BRWD1 and miR-1277 in OA cartilage tissues was analyzed by Pearson’s correlation coefficient analysis. **G** The expression level of circ-BRWD1 in IL-1β, IL-1β+pCD5-ciR or IL-1β+circ-BRWD1 treated CHON-001 cells and control cells was detected by qRT-PCR assay. **H** The level of miR-1277 in IL-1β, IL-1β+si-NC, IL-1β+si-circ-BRWD1, IL-1β+pCD5-ciR, or IL-1β+circ-BRWD1 treated CHON-001 cells and control cells was examined by qRT-PCR assay. The expression of miR-1277 was examined by 2^-ΔΔCt^ method with normalization to U6. *P < 0.05, **P < 0.01, ***P < 0.001, ****P < 0.0001

To explore the underlying mechanisms of circ-BRWD1 in OA development, we searched online website circinteractome and found that miR-1277 contained the complementary sequences of circ-BRWD1 (Fig. 4A). Then dual-luciferase reporter assay and RIP assay were conducted to verify the interaction between circ-BRWD1 and miR-1277. As demonstrated by dual-luciferase reporter assay, miR-1277 transfection strikingly suppressed the luciferase activity of WT-circ-BRWD1 in CHON-001 cells, but had no effect on the luciferase activity of MUT-circ-BRWD1 (Fig. 4B). RIP assay showed that the levels of miR-1277 and circ-BRWD1 immunoprecipitated with Ago2 were all enhanced compared to IgG control groups, further confirming the combination between circ-BRWD1 and miR-1277 (Fig. 4C). Moreover, we found that miR-1277 level was reduced in OA cartilage tissues and different doses of IL-1β-treated CHON-001 cells (Fig. 4D, E). As estimated by Pearson’s correlation coefficient analysis, there was an inverse correlation between miR-1277 and circ-BRWD1 in OA cartilage tissues (Fig. 4F). In addition, the overexpression vector of circ-BRWD1 was successfully transfected in IL-1β-treated CHON-001 cells, as demonstrated by the upregulation of circ-BRWD1 in IL-1β-treated CHON-001 cells after the transfection of circ-BRWD1 (Fig. 4G). We also demonstrated that circ-BRWD1 silencing markedly elevated the expression level of miR-1277 in IL-1β-stimulated CHON-001 cells, while circ-BRWD1 overexpression presented the opposite results (Fig. 4H). Collectively, circ-BRWD1 negatively modulated miR-1277 expression via acting as the sponge for miR-1277.

### Inhibition of miR-1277 abrogated the effects of circ-BRWD1 knockdown on cell viability, apoptosis, inflammation and ECM degradation in IL-1β-treated CHON-001 cells

**Fig. 5:**
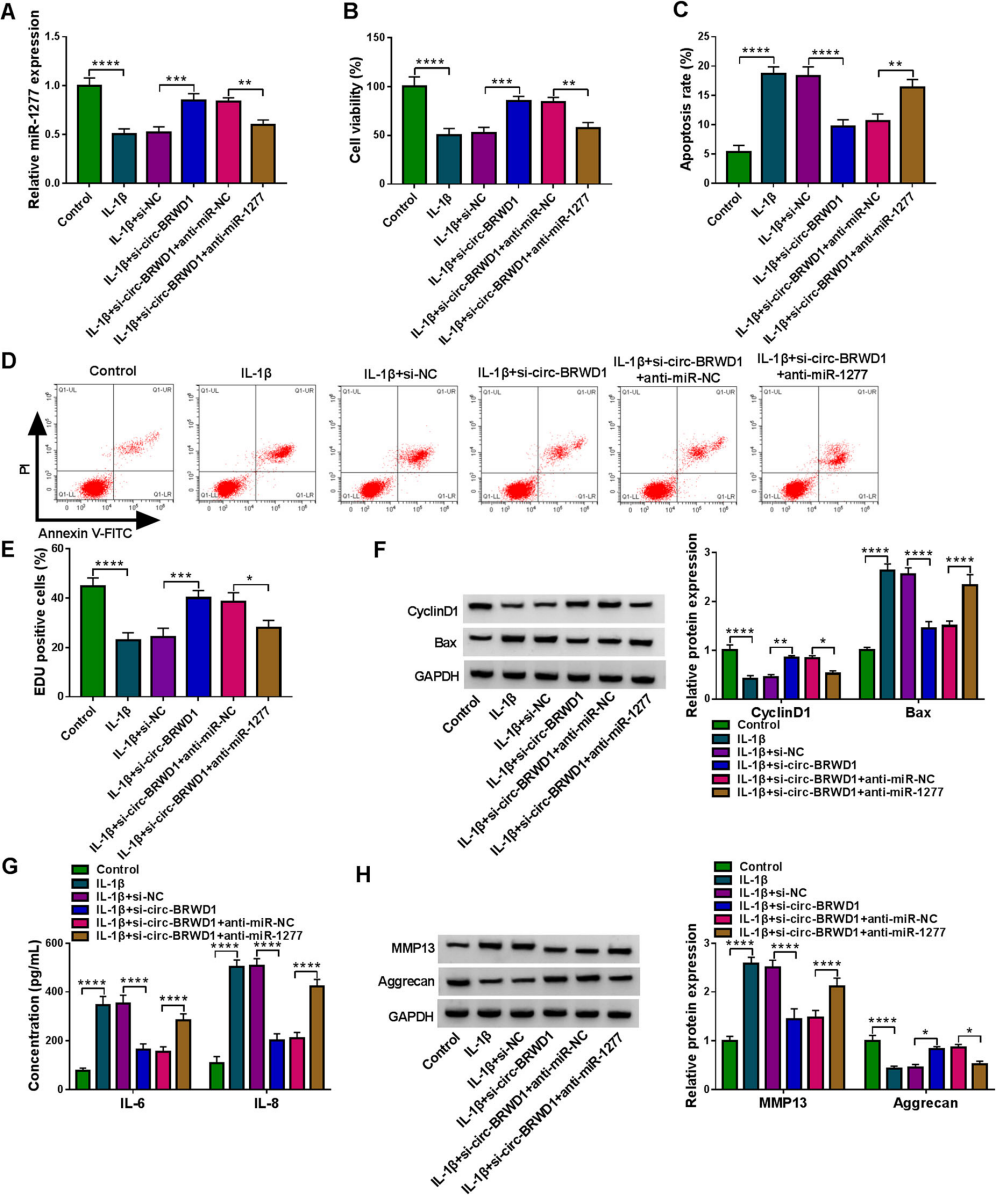
Circ-BRWD1 silencing promoted cell viability and inhibited apoptosis, inflammation and ECM degradation in IL-1β-stimulated CHON-001 cells by binding to miR-1277. CHON-001 cells were assigned to control, IL-1β, IL-1β+si-NC, IL-1β+si-circ-BRWD1, IL-1β+si-circ-BRWD1+anti-miR-NC, and IL-1β+si-circ-BRWD1+anti-miR-1277 groups. **A** QRT-PCR assay was adopted for miR-1277 level in CHON-001 cells. **B**–**D** The viability and apoptosis of CHON-001 cells were evaluated by CCK-8 assay and flow cytometry analysis, respectively. **E** The proliferation of CHON-001 cells was assessed by EDU assay. **F** The protein levels of CyclinD1 and Bax were measured by western blot assay. **G** ELISA was conducted for the levels of IL-6 and IL-8 in CHON-001 cells. **H** Western blot assay was carried out for the protein levels of MMP13 and aggrecan in CHON-001 cells. The expression of miR-1277 was examined by 2^-ΔΔCt^ method with normalization to U6. *P < 0.05, **P < 0.01, ***P < 0.001, ****P < 0.0001

To further analyze the functions of circ-BRWD1/miR-1277 axis in OA progression, CHON-001 cells were divided into 6 groups: control, IL-1β, IL-1β+si-NC, IL-1β+si-circ-BRWD1, IL-1β+si-circ-BRWD1+anti-miR-NC and IL-1β+si-circ-BRWD1+anti-miR-1277. As shown in Fig. 5A, circ-BRWD1 knockdown significantly elevated miR-1277 level in IL-1β-treated CHON-001 cells, while miR-1277 inhibition reversed the influence. The results of CCK-8 assay and flow cytometry analysis indicated that circ-BRWD1 silencing facilitated the viability and repressed the apoptosis of CHON-001 cells mediated by IL-1β treatment, with miR-1277 suppression ameliorated the impacts (Fig. 5B–D). EDU assay showed that that circ-BRWD1 silencing aggravated the viability in IL-1β-treated CHON-001 cells, while miR-1277 inhibition overturned the impact (Fig. 5E). Circ-BRWD1 knockdown upregulated CyclinD1 protein level and downregulated Bax protein level in IL-1β-stimulated CHON-001 cells, with miR-1277 inhibition abrogated the effects (Fig. 5F). ELISA data showed that circ-BRWD1 silencing reduced the concentrations of IL-6 and IL-8 in IL-1β-stimulated CHON-001 cells, whereas these impacts were abolished by decreasing miR-1277 (Fig. 5G). Furthermore, western blot assay showed that MMP13 level was reduced and aggrecan level was elevated in IL-1β-treated CHON-001 cells with circ-BRWD1 silencing, while miR-1277 suppression ameliorated the effects (Fig. 5H). Taken together, circ-BRWD1 interference relieved IL-1β-induced injury of CHON-001 cells by targeting miR-1277.

### TRAF6 was a direct target gene of miR-1277

**Fig. 6:**
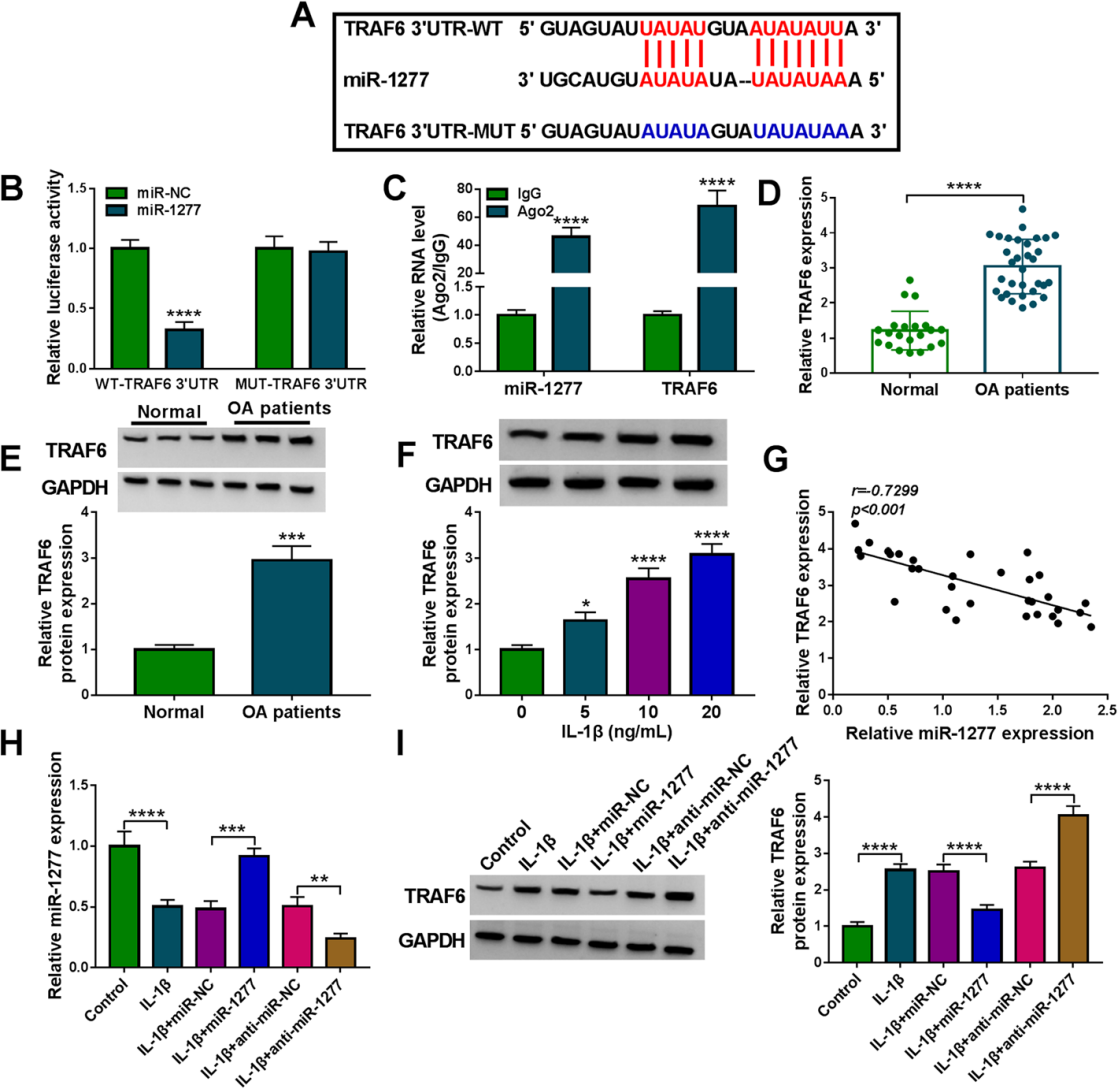
TRAF6 was directly targeted by miR-1277. **A** The predicted binding sites between TRAF6 and miR-1277. **B**, **C** The interaction between miR-1277 and TRAF6 was analyzed by dual-luciferase reporter assay and RIP assay. **D**, **E** The mRNA and protein levels of TRAF6 in OA cartilage tissues and normal cartilage tissues were determined by qRT-PCR assay and western blot assay, respectively. **F** The protein level of TRAF6 in different concentrations of IL-1β-treated CHON-001 cells was measured by western blot assay. **G** The correlation between miR-1277 and TRAF6 in OA cartilage tissues was evaluated by Pearson’s correlation coefficient analysis. **H**, **I** The levels of miR-1277 and TRAF6 protein in IL-1β, IL-1β+miR-NC, IL-1β+miR-1277, IL-1β+anti-miR-NC, or IL-1β+anti-miR-1277 treated CHON-001 cells or untreated CHON-001 cells were measured by qRT-PCR assay and western blot assay, respectively. TRAF6 mRNA expression and miR-1277 expression were examined by 2^-ΔΔCt^ method with normalization to GAPDH and U6, respectively. *P < 0.05, **P < 0.01, ***P < 0.001, ****P < 0.0001

Through analyzing Targetscan software, TRAF6 was found to be a target gene of miR-1277 (Fig. 6A). Then dual-luciferase reporter assay showed that miR-1277 transfection resulted in a remarkable suppression in the luciferase activity of WT-TRAF6 3′UTR in CHON-001 cells, while there was no impact on the luciferase activity in MUT-TRAF6 3′UTR groups (Fig. 6B). Subsequently, RIP assay was conducted to further determine the combination between miR-1277 and TRAF6. The results showed that miR-1277 and TRAF6 levels were all upregulated in Ago2 immunoprecipitates in comparison with IgG groups (Fig. 6C). As we expected, the mRNA and protein levels of TRAF6 were all conspicuously elevated in OA cartilage tissues compared to normal cartilage tissues (Fig. 6D, E). We also determined the protein level of TRAF6 in IL-1β-treated CHON-001 cells, exhibiting that TRAF6 protein level was remarkably raised in IL-1β-treated CHON-001 cells in a dose-dependent way (Fig. 6F). Furthermore, we observed that TRAF6 level was negatively correlated with miR-1277 level in OA cartilage tissues (Fig. 6G). Additionally, our results showed that miR-1277 transfection led to a marked increase in miR-1277 level and anti-miR-1277 transfection led to a distinct decrease in miR-1277 level in IL-1β-treated CHON-001 cells, which indicated the successful transfection of miR-1277 and anti-miR-1277 (Fig. 6H). Western blot assay data showed that miR-1277 overexpression apparently reduced TRAF6 protein level in IL-1β-treated CHON-001 cells, while miR-1277 suppression exhibited the opposite result in TRAF6 protein level (Fig. 6I). Collectively, miR-1277 negatively regulated TRAF6 expression by direct interaction.

### MiR-1277 overexpression promoted cell viability and inhibited apoptosis, inflammation, and ECM degradation in IL-1β-activated CHON-001 cells

**Fig. 7:**
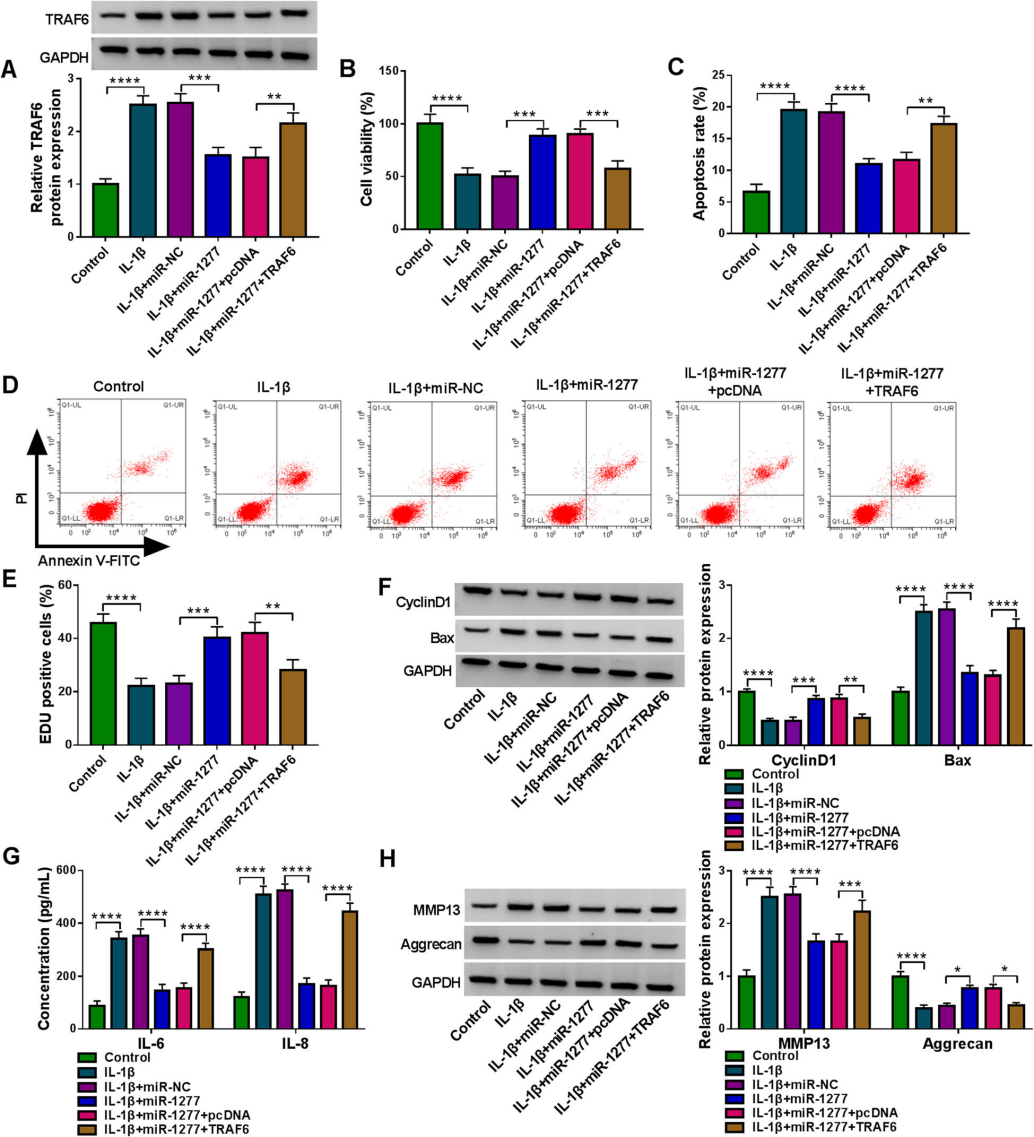
Relationship between miR-1277 and TRAF6 in regulating IL-1β-mediated CHON-001 cell viability, apoptosis, inflammation and ECM degradation. CHON-001 cells were divided into 6 groups: control, IL-1β+miR-NC, IL-1β+miR-1277, IL-1β+miR-1277+pcDNA and IL-1β+miR-1277+TRAF6. **A** Western blot assay was utilized for TRAF6 protein level in each group. **B**–**E** CCK-8 assay, flow cytometry analysis and EDU assay were conducted for the viability, apoptosis and proliferation of CHON-001 cells. **F** The protein levels of CyclinD1 and Bax in CHON-001 cells were measured via western blot assay. **G** ELISA kits were used for the concentrations of IL-6 and IL-8 in CHON-001 cells. **H** Western blot assay was employed for the protein levels of MMP13 and aggrecan in CHON-001 cells. *P < 0.05,**P < 0.01, ***P < 0.001, ****P < 0.0001

To elucidate the association between miR-1277 and TRAF6 in OA development, CHON-001 cells were assigned to 6 groups: control, IL-1β+miR-NC, IL-1β+miR-1277, IL-1β+miR-1277+pcDNA, and IL-1β+miR-1277+TRAF6. As we observed in Fig. 7A, TRAF6 transfection effectively restored the downregulation of TRAF6 protein level mediated by miR-1277 in IL-1β-stimulated CHON-001 cells. As illustrated by CCK-8 assay, flow cytometry analysis and EDU assay, miR-1277 overexpression strikingly facilitated cell viability and proliferation and restrained cell apoptosis in IL-1β-activated CHON-001 cells, while the impacts were rescued by elevating TRAF6 (Fig. 7B–E). Western blot assay showed that miR-1277 overexpression increased CyclinD1 expression and decreased Bax expression in IL-1β-treated CHON-001 cells, while the impacts were reversed by elevating TRAF6 (Fig. 7F). ELISA showed that the concentrations of IL-6 and IL-8 in IL-1β-treated CHON-001 cells were reduced following the upregulation of miR-1277, while TRAF6 overexpression abolished the impacts (Fig. 7G). In addition, miR-1277 overexpression decreased MMP13 level and increased aggrecan level in IL-1β-stimulated CHON-001 cells, with TRAF6 elevation reversing the impacts (Fig. 7H). All results indicated that miR-1277 overexpression attenuated IL-1β-induced injury of CHON-001 cells by targeting TRAF6.

### Circ-BRWD1 knockdown reduced TRAF6 expression via sponging miR-1277

**Fig. 8:**
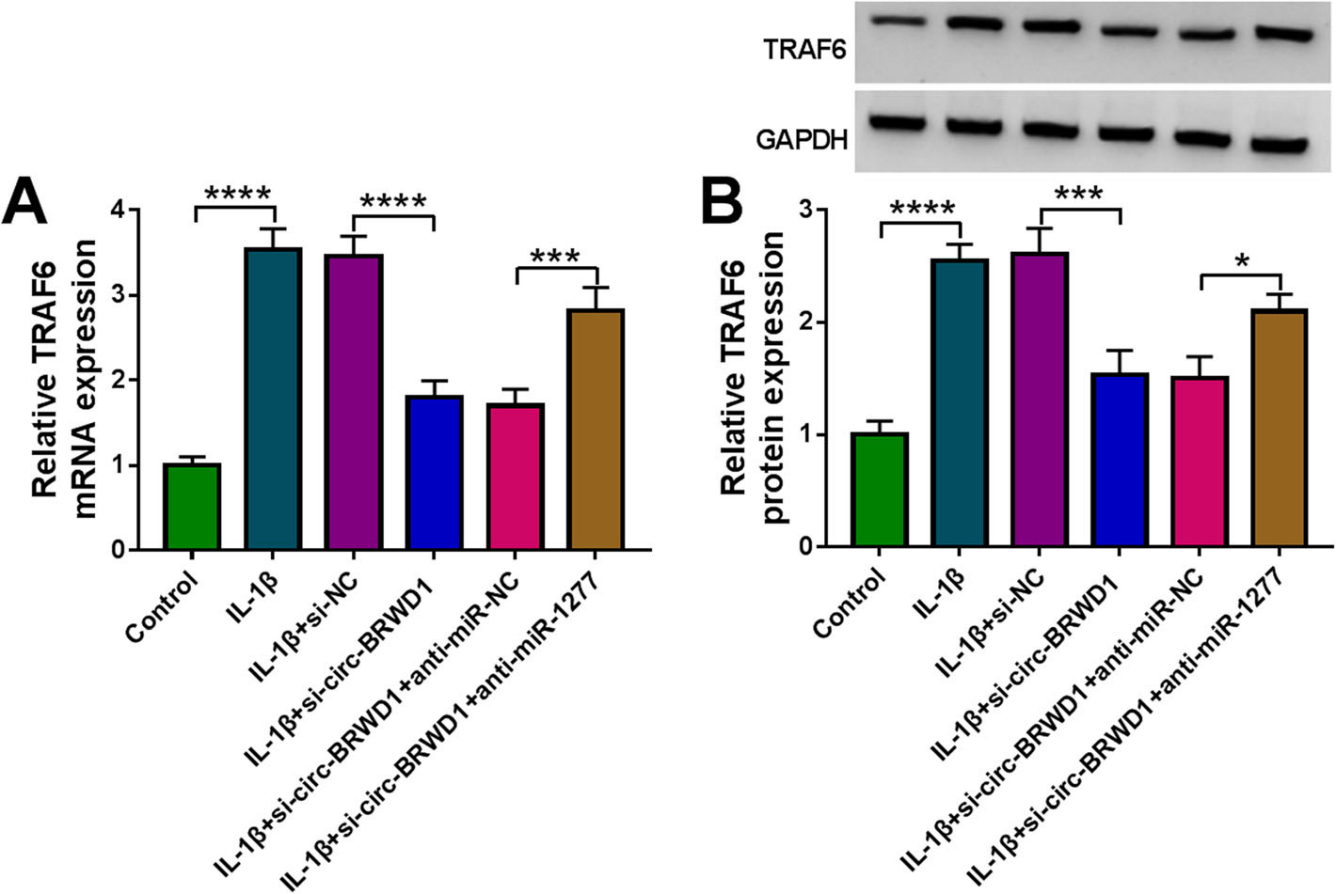
Circ-BRWD1 positively regulated TRAF8 expression by targeting miR-1277 in IL-1β-treated CHON-001 cells. **A**, **B** The mRNA and protein levels of TRAF6 in IL-1β, IL-1β+si-NC, IL-1β+si-circ-BRWD1, IL-1β+si-circ-BRWD1+anti-miR-NC, or IL-1β+si-circ-BRWD1+anti-miR-1277 treated or untreated CHON-001 cells were determined by qRT-PCR assay and western blot assay, respectively. TRAF6 mRNA expression was examined by 2^-ΔΔCt^ method with normalization to GAPDH. *P < 0.05, ***P < 0.001, ****P < 0.0001

Finally, we further analyzed the relationships among circ-BRWD1, miR-1277 and TRAF6 in IL-1β-treated CHON-001 cells. Our results of qRT-PCR assay and western blot assay presented that IL-1β treatment led to marked enhancement in TRAF6 mRNA and protein levels in CHON-001 cells. Moreover, we found that circ-BRWD1 silencing significantly decreased the mRNA and protein levels of TRAF6 in IL-1β-stimulated CHON-001 cells, while miR-1277 suppression ameliorated these impacts (Fig. 8A, B). Taken together, circ-BRWD1 positively modulated TRAF6 expression through functioning as the sponge for miR-1277 in IL-1β-stimulated CHON-001 cells.

## Discussion

Up to date, numerous circRNAs have been identified to be associated with the progression of human diseases. Nevertheless, the biological roles of circRNAs in OA are not well known. In the research, we focused on the effects of exosomal circ-BRWD1 in OA pathogenesis. IL-1β was utilized to treat CHON-001 cells to construct an OA model in vitro, as previously mentioned [20, 21]. We found that IL-1β treatment repressed CHON-001 cell viability and induced apoptosis, inflammation and ECM degradation. Moreover, exosomal circ-BRWD1 level was elevated in IL-1β-activated CHON-001 cells. Circ-BRWD1 knockdown protected CHON-001 cells from IL-1β-activated injury by miR-1277/TRAF6 axis.

The vital roles of exosome-mediated circRNAs have been demonstrated in human illnesses. For instance, exosomal circ_MMP2 accelerated the malignancy of hepatocellular carcinoma by modulating miR-136-5p/MMP2 axis [22]. Exosome-mediated circ_0044516 contributed to prostate cancer carcinogenesis [23]. Moreover, several circRNAs, such as circ_0045714 [10], circ_0136474 [11] and circGCN1L1 [24], have been reported to play vital roles in OA. Herein, we elucidated the role of exosome-mediated circ-BRWD1 in OA progression. Circ-BRWD1 level was raised in OA cartilage tissues and IL-1β-induced CHON-001 cells. Circ-BRWD1 level was also increased in the exosomes derived from IL-1β-stimulated CHON-001 cells. Moreover, exosome treatment elevated circ-BRWD1 level in IL-1β-activated CHON-001 cells, while GW4869 treatment reduced circ-BRWD1 level, indicating exosomes mediated the transmission of circ-BRWD1. Functionally, deficiency of circ-BRWD1 promoted cell viability and impeded cell apoptosis and inflammatory factors release mediated by IL-1β in CHON-001 cells. In addition, we measured the levels of MMP13 and aggrecan, which play crucial roles in cartilage ECM production during OA development [25, 26]. Our results exhibited that circ-BRWD1 silencing decreased MMP13 level and increased aggrecan level in IL-1β-stimulated CHON-001 cells, indicating ECM degradation was inhibited. Taken together, exosomal circ-BRWD1 knockdown blocked OA development.

Subsequently, the potential mechanisms of circ-BRWD1 in regulating OA progression were explored. The data indicated that circ-BRWD1 served as the sponge for miR-1277 to positively alter TRAF6 expression. MiR-1277 was found to be decreased in OA tissues and IL-1β-stimulated chondrocytes, and its overexpression restrained the degradation of ECM [17]. Consistently, we observed that miR-1277 was weakly expressed in OA cartilage tissues and IL-1β-activated CHON-001 cells. Moreover, miR-1277 overexpression promoted cell viability and hampered apoptosis, inflammatory response and ECM degradation in IL-1β-activated CHON-001 cells. MiR-1277 suppression alleviated the impacts of circ-BRWD1 deficiency on cell viability, apoptosis, inflammation and ECM degradation in IL-1β-activated CHON-001 cells, suggesting that circ-BRWD1 deficiency blocked OA progression by sponging miR-1277. Additionally, TRAF6 was identified to be the target gene of miR-1277 for the first time, though it could be targeted by multiple miRNAs [27–29]. Of note, we demonstrated that TRAF6 upregulation abrogated the impact of miR-1277 on the progression of IL-1β-activated CHON-001 cells, indicating that miR-1277 regulated OA development by targeting TRAF6.

## Conclusions

**Fig. 9:**
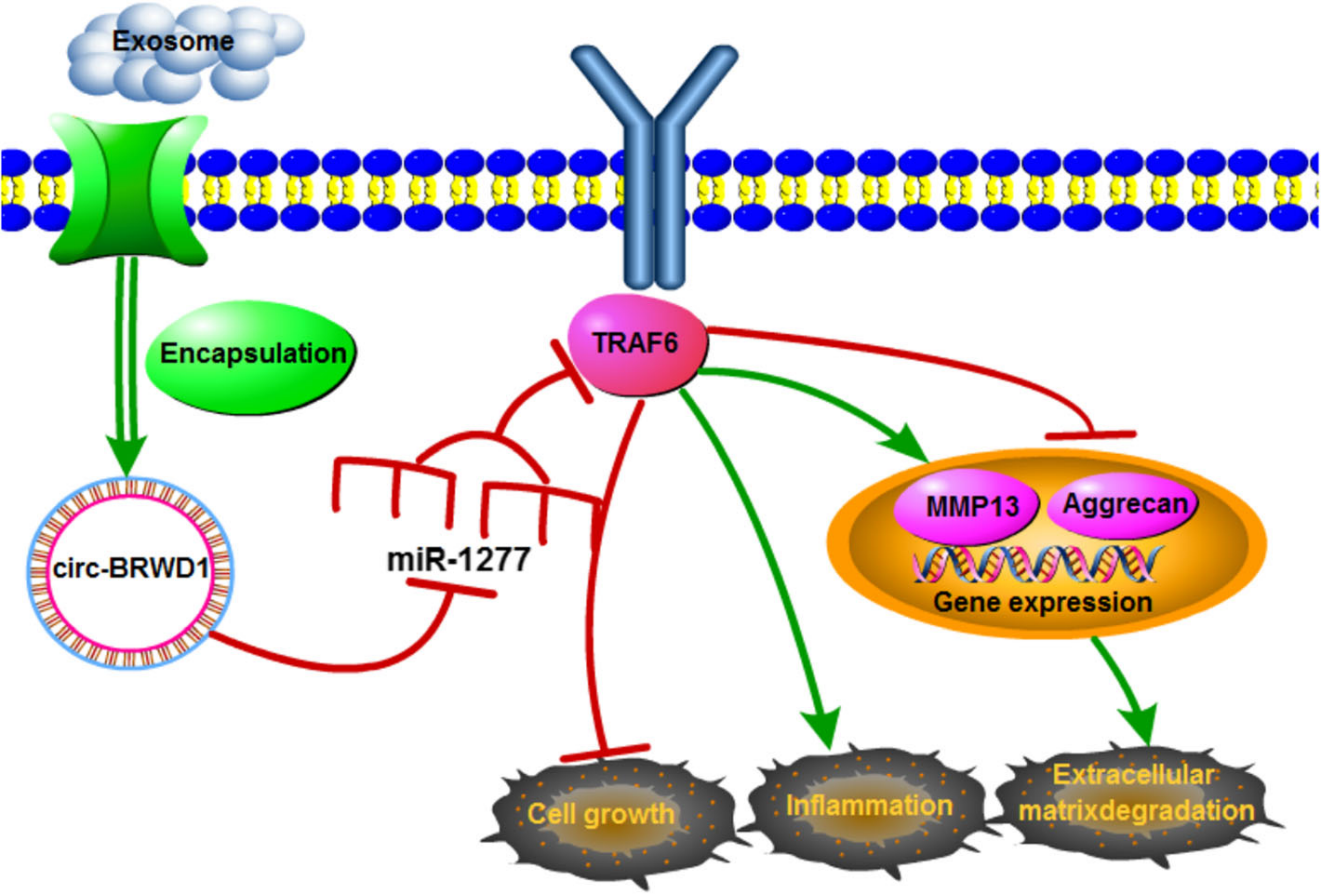
The schematic diagram of exosomal circ-BRWD1 in regulating chondrocyte growth, inflammation, and ECM degradation

Exosomal circ-BRWD1 was upregulated in OA cell model and impeded chondrocyte viability and facilitated apoptosis, inflammation and ECM degradation by elevating TRAF6 via sponging miR-1277 (Fig. 9). The research might bring a new sight for OA therapy.

## Data Availability

The analyzed data sets generated during the present study are available from the corresponding author on reasonable request.

## Abbreviations

OA: Osteoarthritis
CircRNAs: Circular RNAs
BRWD1: Bromodomain and WD repeat domain containing 1
IL-1β: Interleukin-1β
TRAF6: TNF receptor-associated factor 6
TEM: Transmission electron microscopy
CCK-8: Cell Counting Kit-8
EDU: 5-Ethynyl-2′-deoxyuridine
ELISA: Enzyme-linked immunosorbent assay
IL-6: Interleukin-6
IL-8: Interleukin-8
RIP: RNA immunoprecipitation
ECM: Extracellular matrix
ncRNAs: Non-coding RNAs
miRNAs: MicroRNAs
IGF1R: Insulin-like growth factor 1 receptor
3′UTR: 3′ untranslated region
MMP13: Matrix Metallopeptidase 13
ADAMTS5: ADAM metallopeptidase with thrombospondin type 1 motif 5
ATCC: American Type Culture Collection
DMEM: Dulbecco’s modified Eagle’s medium
FBS: Fetal bovine serum
qRT-PCR: Quantitative real-time polymerase chain reaction
GAPDH: Glyceraldehyde 3-phosphate dehydrogenase
RIPA: Radio Immunoprecipitation Assay
BCA: Bicinchoninic acid
ECL: Enhanced chemiluminescence
Annexin V-FITC: Annexin V-fluorescein isothiocyanate
PI: Propidium iodide
IgG: Immunoglobulin G
Ago2: Argonaute-2
MMP2: Matrix Metallopeptidase 2
